# Mortality Prediction in Cardiac Intensive Care Unit Patients: A Systematic Review of Existing and Artificial Intelligence Augmented Approaches

**DOI:** 10.3389/frai.2022.876007

**Published:** 2022-05-31

**Authors:** Nikita Rafie, Jacob C. Jentzer, Peter A. Noseworthy, Anthony H. Kashou

**Affiliations:** ^1^Department of Medicine, Mayo Clinic, Rochester, MN, United States; ^2^Division of Pulmonary and Critical Care Medicine, Department of Medicine, Mayo Clinic, Rochester, MN, United States; ^3^Department of Cardiovascular Medicine, Mayo Clinic, Rochester, MN, United States

**Keywords:** electrocardiogram, ECG interpretation, artificial intelligence, mortality prediction, machine learning, cardiac intensive care unit

## Abstract

The medical complexity and high acuity of patients in the cardiac intensive care unit make for a unique patient population with high morbidity and mortality. While there are many tools for predictions of mortality in other settings, there is a lack of robust mortality prediction tools for cardiac intensive care unit patients. The ongoing advances in artificial intelligence and machine learning also pose a potential asset to the advancement of mortality prediction. Artificial intelligence algorithms have been developed for application of electrocardiogram interpretation with promising accuracy and clinical application. Additionally, artificial intelligence algorithms applied to electrocardiogram interpretation have been developed to predict various variables such as structural heart disease, left ventricular systolic dysfunction, and atrial fibrillation. These variables can be used and applied to new mortality prediction models that are dynamic with the changes in the patient's clinical course and may lead to more accurate and reliable mortality prediction. The application of artificial intelligence to mortality prediction will fill the gaps left by current mortality prediction tools.

## Introduction

The modern cardiac intensive care unit (CICU) has evolved from the original coronary care unit (CCU) paradigm developed 50 years ago to provide more effective care for patients with acute myocardial infarction (AMI) (Katz et al., 2010) into an integrated, multidisciplinary unit that provides comprehensive care to an increasingly diverse and complex patient population. Recent studies highlight the dramatic changes in the contemporary CICU patient population including a shift from AMI to circulatory failure and multi-organ dysfunction (Katz et al., 2010; Bohula et al., 2019; Jentzer et al., 2019c). These studies underscore the substantial complexity of the modern CICU population and demonstrate the multitude of acute and chronic cardiac and non-cardiac illnesses suffered by these patients (Katz et al., 2010; Bohula et al., 2019; Jentzer et al., 2019c; Lyle et al., 2020; Miller et al., 2021). There has been a recent emphasis on outcome prognostication and mortality risk stratification in CICU patients (Jentzer and Rossello, 2021). Risk stratification strategies tailored to this medically complex patient cohort are necessary to direct management to improve patient care and reduce mortality (Miller et al., 2021).

The breadth of diagnostic testing performed in the CICU (e.g., laboratory and vital sign assessment, cardiac telemetry, invasive, and non-invasive hemodynamic monitoring) provides an enormous quantity of data. Previous CICU mortality prediction studies have failed to fully leverage the complexity of the data available in the medical record to optimize prediction performance. This has created a niche for the use of artificial intelligence (AI) methods to integrate variables to provide risk predictions. For instance, digitized electrocardiographic data has been used as a substrate to create deep learning models capable of diagnostic predictions. By treating the standard 12-lead electrocardiogram (ECG) as an image and applying deep learning neural networks to the cardiac biosignal, AI models can discern the presence or absence of specific underlying cardiac diseases (Attia et al., 2019a; Tison et al., 2019; Christopoulos et al., 2020; Gladding et al., 2020; Jentzer et al., 2021a; Kashou et al., 2021). This diagnostic and prognostic capability may be particularly valuable to the care of patients in the CICU. In this systematic review, we explore (i) existing CICU mortality prediction models, (ii) how AI-ECG can be embedded into clinical practice, and (iii) use of AI-ECG in CICU for mortality prediction.

## Methods

We conducted a thorough literature search using our institutional database search which includes PubMed, UpToDate, Catalog, and Google Scholar. Keywords used for the search were: “artificial intelligence,” “machine learning,” “deep neural network,” “mortality prediction,” “risk score,” “cardiac intensive care unit,” “CICU,” “CCU,” and “ECG.” Articles were filtered to include original studies and meta-analyses. There is limited work in this field as it relates to AI-ECG, and there is even less literature in a CICU population. The authors made a comprehensive review of the limited literature available.

## Existing CICU Mortality Prediction Models

Given the high mortality and complexity of patients in the CICU, a mortality risk stratification tool could guide clinical decision making during and after hospitalization. Multiple mortality prediction models for critically ill patients have been developed in non-CICU settings, including medical and surgical intensive care units (ICUs). However, these prediction models were not developed specifically for use in the CICU and therefore are poorly calibrated for such patients. Common risk scores in the ICU include the Acute Physiology and Chronic Health Evaluation (APACHE)-III, APACHE-IV, Sequential Organ Failure Assessment (SOFA), and Oxford Acute Severity of Illness Score (OASIS) (Table 1). Analysis of these risk scores demonstrate inconsistent performance for mortality risk stratification when applied to the CICU population characterized by good discrimination but poor calibration (Jentzer et al., 2019a, 2020; Jentzer and Rossello, 2021; Miller et al., 2021). The need for a CICU-specific mortality prediction tool led to the development of the Mayo Cardiac Intensive Care Unit Admission Risk Score (M-CARS), which is the first published risk score designed for use in CICU patients and outperforms standard severity of illness scores for prediction of in-hospital mortality in terms of both discrimination and calibration (Jentzer et al., 2019a).

**Table 1:** Commonly used risk scores: APACHE-III, APACHE-IV, SOFA, and OASIS.

**APACHE-III Score** (0–299) (Knaus et al., 1991).		
**Variable**	**Points assigned**	
**Age**	**0–24**	
**Comorbid condition**	**0–23**	
**Physiology**	**0–252**	
**APACHE-IV Score – 129 Variables from the worst values from the initial 24 h of intensive care unit (ICU) admission (Zimmerman et al.**, **2006****)**.		
**SOFA** score calculated on admission and every 48 h until discharge. Initial and highest scores of more than 11 or mean scores of more than five correspond to a mortality of more than 80% (Ferreira et al., 2001).		
**Variable**	**Value**	**Points assigned**
PaO_2_/FiO_2_	>400	0
	301–400	1
	≤ 300	2
	101–200 with ventilatory support	3
	≤ 100 with ventilatory support	4
Platelets	>150 × 10^3^/mm^3^	0
	101–150 × 10^3^/mm^3^	1
	51–100 × 10^3^/mm^3^	2
	21–50 × 10^3^/mm^3^	3
	≤ 20 × 10^3^/mm^3^	4
Bilirubin	<1.2 mg/dL	0
	1.2–1.9 mg/dL	1
	2–5.9 mg/dL	2
	6–11.9 mg/dL	3
	>12 mg/dL	4
Blood Pressure	Hypotension absent	0
	Mean arterial pressure <70 mmHg	1
	On dopamine ≤ 5 mcg/kg/min or any dobutamine	2
	On dopamine >5 mcg/kg/min, epinephrine ≤ 0.1 mcg/kg/min, or norepinephrine ≤ 0.1 mcg/kg/min	3
	On dopamine >15 mcg/kg/min, epinephrine >0.1 mcg/kg/min, or norepinephrine >0.1 mcg/kg/min	4
Glasgow Coma Score	15	0
	13–14	1
	10–12	2
	6–9	3
	<6	4
Creatinine	<1.2 mg/dL	0
	1.2–1.9 mg/dL	1
	2–3.4 mg/dL	2
	3.5–4.9 mg/dL or urine output 200–500 mL/day	3
	>5 mg/dL or urine output <200 mL/day	4
**OASIS** scores in the 20th decile correlated with an 18% ICU and 25% hospital mortality rate and Oasis scores in the 30th decile correlated with a 40% ICU and 49% hospital mortality rate (Johnson et al., 2013).		
Pre-ICU Length of Stay	<0.17 h	5
	0.17–4.94 h	3
	4.95–24 h	0
	24.01–311.8 h	2
	>311.8 h	1
Age	<24 years	0
	24–53 years	3
	54–77 years	6
	78–89 years	9
	>90 years	7
Glasgow coma score	3–7	10
	8-13	4
	14	3
	15	0
Heart rate	<33 beats per minute	4
	33–88 beats per minute	0
	89–106 beats per minute	1
	107–125 beats per minute	3
	>125 beats per minute	6
Mean arterial pressure	<20.65 mmHg	4
	20.65–50.99 mmHg	3
	51–61.32 mmHg	2
	61.33–143.44 mmHg	0
	>143.44 mmHg	3
Respiratory rate	<6 breaths per minute	10
	6–12 breaths per minute	1
	13–22 breaths per minute	0
	23–30 breaths per minute	1
	31–44 breaths per minute	6
	>44 breaths per minute	9
Temperature	<33.22°C	3
	33.22–35.93°C	4
	35.94–36.39°C	2
	36.4–36.88°C	0
	36.89–39.88°C	2
	>39.88	6
Urine output	<671 cc/day	10
	671–1426.99 cc/day	5
	1427–253.99 cc/day	1
	2,544–6,896 cc/day	0
	>6,896 cc/day	8
Ventilated	No	0
	Yes	9
Elective surgery	No	6
	Yes	0

M-CARS incorporates data available at the time of CICU admission to predict in-hospital mortality using 7 clinical variables (Jentzer et al., 2019a). The component variables include blood urea nitrogen (BUN), anion gap, Braden skin score, red cell distribution width (RDW), diagnosis of cardiac arrest, diagnosis of shock, and diagnosis of respiratory failure (Table 2). Variables are collected at the time of CICU admission with a score ranging from 0 to 10. In addition, the M-CARS on CICU admission can predict the likelihood of requiring critical care interventions during the CICU stay (Breen et al., 2021a). An M-CARS <2 correlates with a patient that typically does not require CICU level of care based on low mortality (<1% in hospital) and infrequent need for critical care resources (Jentzer et al., 2019a; Breen et al., 2021a). A score >6 is associated with a high in-hospital mortality risk that generally exceeds 50%, with high resource utilization (Jentzer et al., 2019a; Breen et al., 2021a). Knowing these risks early in the patient's hospital course will allow for additional interventions and collaborations, such as with palliative care. Notably, an increasing M-CARS is associated with incrementally higher all-cause mortality out to 1 year after CICU admission, including among patients surviving to hospital discharge (Breen et al., 2021c). These findings highlight the multitude of ways that the prognostic information captured by the M-CARS can be incorporated into clinical decision-making.

**Table 2:** Calculating the Mayo Cardiac Intensive Care Unit Admission Risk Score (M-CARS). Calculated at time of admission; score ranges from 0 to 10.

**Variable**	**Value**	**Points assigned**
Admission value of BUN	>23 mg/dL	1
	≤ 23 mg/dL	0
Admission value of anion gap	>14 mmol/L	1
	≤ 14 mmol/L	0
Admission Braden skin score	≤ 12	2
	13–15	1
	>15	0
Admission value of RDW	>14.3%	1
	≤ 14.3%	0
Admission diagnosis of cardiac arrest	Yes	2
	No	0
Admission diagnosis of shock	Yes	2
	No	0
Admission diagnosis of respiratory failure	Yes	1
	No	0

As emphasized by M-CARS, abnormalities in common laboratory tests at the time of CICU admission have been associated with higher mortality, including hyponatremia, hyperkalemia, hypochloremia, hypoalbuminemia, anemia, elevated RDW, elevated anion gap, and elevated BUN (Jentzer et al., 2019a; Breen et al., 2020, 2021b; Rayes et al., 2020; Padkins et al., 2021). One variable utilized in the M-CARS requires further clarification—the Braden skin score, which is a bedside nursing tool used to predict the risk of skin pressure injury. The Braden skin score ranges from six to 23 and is derived from six individual subscores reflecting different components of risk; a lower score predicts an increased risk of pressure injury (Table 3). Prior studies have demonstrated that a lower Braden skin score at the time of CICU admission is associated with a higher risk of all-cause mortality (Bergstrom et al., 1987; Jentzer et al., 2019b). A lower Braden skin score is a proposed marker of frailty, making the M-CARS novel insofar as it is the first mortality risk score to include this important construct (Jentzer et al., 2019a,b).

**Table 3:** Braden scale for predicting risk of pressure-induced injury.

**Variable**	**Value**	**Points assigned**
Sensory perception	Completely limited	1
	Very limited	2
	Slightly limited	3
	No impairment	4
Moisture	Constantly moist	1
	Very moist	2
	Occasionally moist	3
	Rarely moist	4
Activity	Bedfast	1
	Chairfast	2
	Walks occasionally	3
	Walks frequently	4
Mobility	Completely immobile	1
	Very limited	2
	Slightly limited	3
	No limitation	4
Nutrition	Very poor	1
	Probably inadequate	2
	Adequate	3
	Excellent	4
Friction and Shear	Problem	1
	Potential problem	2
	No apparent problem	3

*Score of 18 or less indicates high risk of developing pressure sores (Bergstrom et al., 1987)*.

Prior to the establishment of M-CARS, mortality prediction tools used in the CICU were disease specific and not necessarily formulated for the broader CICU patient population. The Thrombolysis in Myocardial Infarction (TIMI), the Global Registry of Acute Coronary Events (GRACE), and Zwolle risk scores were developed for patients with AMI or ACS (Antman et al., 2000; Granger et al., 2003; De Luca et al., 2004) (Table 4). In patients with acute decompensated HF, several risk scores help predict short-term mortality. Some of these include the Enhanced Feedback for Effective Cardiac Treatment (EFFECT), the Organized Program to Initiate Lifesaving Treatment in Hospitalized Patients with Heart Failure (OPTIMIZE), and the Get with the Guidelines—Heart Failure (GWTG-HF) risk scores (Lee et al., 2003; Abraham et al., 2008; Peterson et al., 2010; Lyle et al., 2020) (Table 5). These scores are capable of providing risk stratification amongst unselected CICU patients; however, their predictive performance is inferior to standard ICU risk scores when applied to CICU patients, even in those with HF (Lyle et al., 2020).

**Table 4:** The TIMI, GRACE, and Zwolle risk scores for prediction of outcome in acute MI/ACS patients.

Higher **TIMI** risk score correlates with increased number of events at 14 day including, all-cause mortality, new or recurrent MI, or severe recurrent ischemia requiring revascularization (Antman et al., 2000).		
**Variable**	**Value**	**Points assigned**
Age	≥65	1
	<65	0
Presence of at least three risk factors for coronary heart disease	Yes	1
	No	0
Prior coronary stenosis of ≥50%	Yes	1
	No	0
Presence of ST segment deviation on admission ECG	Yes	1
	No	0
At least two anginal episodes in prior 24 h	Yes	1
	No	0
Elevated serum cardiac biomarkers	Yes	1
	No	0
Use of aspirin in prior seven day	Yes	1
	No	0
A low **GRACE** score ( ≤ 108) correlated with <1% in-hospital death, an intermediate GRACE score (109–140) correlated with 1–3% in-hospital death, and a high GRACE score (>140%) correlated with >3% in-hospital death (Granger et al., 2003).		
**Variable**	**Value**	**Points assigned**
Age	<40	0
	40–49	18
	50–59	36
	60–69	55
	70–79	73
	80–89	91
	≥90	100
Resting heart rate	<50 beats per minute	0
	50–69.9 beats per minute	3
	70–89.9 beats per minute	9
	90–109.9 beats per minute	14
	110–149.9 beats per minute	23
	150–199.9 beats per minute	35
	≥200 beats per minute	43
Systolic blood pressure	≤ 79.9 mmHg	24
	80–99.9 mmHg	22
	100–119.9 mmHg	18
	120–139.9 mmHg	14
	140–159.9 mmHg	10
	160–199.9 mmHg	4
	≥200 mmHg	0
Initial serum creatinine	0–0.39 mg/dL	1
	0.4–0.79 mg/Dl	3
	0.8–1.19 mg/dL	5
	1.2–1.59 mg/dL	7
	1.6–1.99 mg/dL	9
	2.0–3.99 mg/dL	15
	≥4 mg/dL	20
Other factors	History of heart failure	24
	History of MI	12
	ST segment depression	11
	Elevated cardiac enzymes	15
	No in-hospital percutaneous coronary intervention done	14
Low risk (**Zwolle** score ≤ 3) patients undergoing primary percutaneous coronary intervention had a mortality rate of 0.1% at 2 days and 0.2% between two and 10 days post-MI (De Luca et al., 2004).		
**Variable**	**Value**	**Points assigned**
Killip class	1	0
	2	4
	3–4	9
TIMI flow post intervention	3	0
	2	1
	0–1	2
Age	<60	0
	≥60	2
Three-vessel disease	No	0
	Yes	1
Anterior infarction	No	0
	Yes	1
Ischemia time	≤ 4 h	0
	>4 h	1

**Table 5:** The EFFECT, OPTIMIZE-HF, and GWTG-HF risk scores predict outcomes in patients with acute decompensated heart failure.

A low risk **EFFECT** score of <60 corresponds to 0.4% 30-day and 7.8% 1-year mortality. A high risk **EFFECT** score of >150 corresponds to 59% 30-day and 78.8% 1-year mortality (Lee et al., 2003).		
**Variable**	**30-day score**	**1-year score**
Age	+age (in years)	+age (in years)
Respiratory rate	+rate (in breaths/minute)	+rate (in breaths/minute)
Systolic blood pressure ≥180 mmHg	−60	−50
160–179 mmHg	−55	−45
140–159 mmHg	−50	−40
120–139 mmHg	−45	−35
100–119 mmHg	−40	−30
90–99 mmHg	−35	−25
<90 mmHg	−30	−20
Urea nitrogen (maximum 60 mg/dL)	+level (in mg/dL)	+level (in mg/dL)
Sodium concentration <136 mEq/L	+10	+10
Cerebrovascular disease	+10	+10
Dementia	+20	+15
Chronic obstructive pulmonary disease	+10	+10
Hepatic cirrhosis	+25	+35
Cancer	+15	+15
Hemoglobin <10.0 g/dL	NA	+10
The score obtained using the **OPTIMIZE**-**HF** risk score is directly associated with the probability of in-hospital mortality. A score of 70 correlates with 50% mortality and a score of 90 correlates with 90% mortality (Abraham et al., 2008).		
**Variable**	**Value**	**Points assigned**
Age	20	0
	25	2
	30	3
	35	5
	40	6
	45	8
	50	9
	55	11
	60	13
	65	14
	70	16
	75	17
	80	19
	85	20
	90	22
	95	24
Heart rate (beats/min)	65	0
	70	1
	75	1
	80	2
	85	3
	90	4
	95	4
	100	5
	105	6
	110	6
Systolic blood pressure (mmHg)	50	22
	60	20
	70	18
	80	16
	90	14
	100	12
	110	10
	120	8
	130	6
	140	4
	150	2
	160	0
Sodium (mEq/L)	110	13
	115	11
	120	9
	125	7
	130	4
	135	2
	140	0
	145	2
	150	4
	155	6
	160	8
	165	10
	170	12
Serum creatinine (mg/dL)	0	0
	0.5	2
	1	5
	1.5	7
	2	10
	2.5	12
	3	15
	3.5	17
Primary cause of admission	Heart failure	0
	Other	3
LVSD	No	0
	Yes	1
The greater the **GWTG-HF** risk score, the greater the probability of death. A score of ≥79 correlates with a >50% mortality (Peterson et al., 2010).		
**Variable**	**Value**	**Points assigned**
Age	≤ 19	0
	20–29	3
	30–39	6
	40–49	8
	50–59	11
	60–69	14
	70–79	17
	80–89	19
	90–99	22
	100–109	25
	≥110	28
Heart rate (beats/min)	≤ 79	0
	80–84	1
	85–89	3
	90–94	4
	95–99	5
	100–104	6
	≥105	8
Systolic blood pressure (mmHg)	50–59	28
	60–69	26
	70–79	24
	80–89	23
	90–99	21
	100–109	19
	110–119	17
	120–129	15
	130–139	13
	140–149	11
	150–159	9
	160–169	8
	170–179	6
	180–189	4
	190–199	2
	≥200	0
Sodium (mEq/L)	≤ 130	4
	131–133	3
	134–136	2
	137–138	1
	≥139	0
Blood urea nitrogen	≤ 9	0
	10–19	2
	20–29	4
	30–39	6
	40–49	8
	50–59	9
	60–69	11
	70–79	13
	80–89	15
	90–99	17
	100–109	19
	110–119	21
	120–129	23
	130–139	25
	140–149	27
	≥150	28
Black race	Yes	0
	No	3
Chronic obstructive pulmonary disease	Yes	2
	No	0

## AI-ECG in Clinical Practice

The surface ECG is one of the most ubiquitous point-of-care tests in contemporary CICU practice and remains the gold standard for diagnosing a variety of clinical syndromes. As such, leveraging the ECG to provide an assessment of a patient's cardiac function would be a powerful tool in the CICU. Accordingly, recently developed AI-ECG algorithms can identify various patient characteristics and disease processes based on the surface 12-lead ECG alone. AI-ECG models have demonstrated the ability to classify age and sex, as well as the ability to detect the presence of atrial fibrillation (AF), left ventricular systolic dysfunction (LVSD), and structural heart diseases (e.g., aortic stenosis, amyloidosis, pulmonary arterial hypertension, mitral valve prolapse, and hypertrophic cardiomyopathy) (Attia et al., 2019a; Tison et al., 2019; Christopoulos et al., 2020; Gladding et al., 2020; Jentzer et al., 2021a; Kashou et al., 2021). While these AI-ECG algorithms were developed based on large cohorts consisting primarily of outpatients, there is no reason to suspect that they would not perform well in CICU patients.

Indeed, a previously developed AI-ECG algorithm can predict the risk of LVSD in the CICU population (Jentzer et al., 2021a). The AI-ECG algorithm used in this study to detect LVSD was derived and validated in almost 100,000 patients from Mayo Clinic (Attia et al., 2019b). The AI-ECG algorithm was developed by using the Keras framework with a Tensorflow backend and Python (Attia et al., 2019b). Half of the sample of the large data set was used to train the network and the remainder of the data set to test the network (Attia et al., 2019b). The original model had a sensitivity of 86.3%, specificity of 85.7%, and accuracy of 85.7% in the validation cohort including a broad patient population (Attia et al., 2019b). In a secondary validation study, 5,680 unique CICU patients with a mean age of 68 ± 15 years (34% females) were included if they underwent an AI-ECG and transthoracic echocardiogram within seven days and were not included in the original derivation cohort. The AI-ECG algorithm was applied to identify the probability of the presence of LVSD, as defined by a left ventricular ejection fraction (LVEF) of ≤ 40% in the CICU cohort and ≤ 35% in the original derivation cohort, which was present in 34% of the CICU patient population as compared to 19.9% for the general population in which the algorithm was derived and validated (Attia et al., 2019b; Jentzer et al., 2021a). The AI-ECG had a sensitivity of 73%, specificity of 78%, negative prediction value of 85%, and overall accuracy of 76% for identifying LVSD in the CICU population (Jentzer et al., 2021a). This was the first study to use AI-ECG to predict LVSD in the CICU patient population and it confirmed the ability of the algorithm to do so, albeit with a slightly reduced accuracy in the setting of a higher pre-test probability of LVSD. One CICU subgroup that demonstrated lower performance of the AI-ECG algorithm was those admitted with ACS (including those who underwent PCI), presumably due to the dynamic changes in both the ECG and LV function by TTE due to myocardial stunning and recovery (Jentzer et al., 2021a). In this population, there was a lower probability of LVSD by the AI-ECG algorithm despite similar mean LVEF compared to the CICU population without ACS leading to a higher rate of false-negative AI-ECG for LVSD emphasizing the need to perform TTE when reduced LVEF is suspected in ACS patients (Jentzer et al., 2021a). Additionally, the inverse correlation between the AI-ECG predicted probability of LVSD and LVEF was stronger in patients without ACS than those with ACS (Jentzer et al., 2021a). It was hypothesized that this population has dramatic dynamic ECG changes that may interfere with the AI-ECG algorithm to accurately predict LVSD (Jentzer et al., 2021a).

In a follow-up study, CICU patients with AF at the time of TTE were examined to determine whether the presence of AF influences the ability of the AI-ECG to identify LVSD. This study again used the previously derived AI-ECG algorithm applied to CICU patients undergoing AI-ECG prediction of LVSD and TTE in the same day for whom data regarding cardiac rhythm was also available (Kashou et al., 2021). The AI-ECG algorithm proved to have an area under the curve (AUC) for identification of LVSD of 0.79 in patients in AF and 0.82 in patients in sinus rhythm (Kashou et al., 2021). When used to detect LVSD in patients in AF, the AI-ECG model had an overall accuracy of 71.4%, sensitivity of 77.5%, specificity of 67.4%, positive predictive value of 62.6%, and negative predictive value of 81.0% (Kashou et al., 2021). When used to detect LVSD in patients in sinus rhythm, the AI-ECG model had an overall accuracy of 76.4%, sensitivity of 67.3%, specificity of 80.4%, positive predictive value of 60.7%, and negative predictive value of 84.5% (Kashou et al., 2021). Nonetheless, this algorithm and application to the CICU population pose a great advancement to the ability to use AI-ECG in the CICU setting. Furthermore, the prediction of LVSD can be used in various mortality prediction tools to better predict mortality throughout the patient's hospitalization as a dynamic variable that can be quickly and easily obtained.

The ability of the AI-ECG to predict LVSD without a formal echocardiogram can be used to triage and treat certain patient populations with suspected LVSD, particularly if they do not have ACS. This may influence decisions regarding specific treatments, such as the rate-control drug option for patients with AF help to identify patients requiring an expedited formal echocardiogram or detailed point of care cardiac ultrasound. In many institutions, a timely echocardiogram cannot be obtained, especially during off-hours when resources are limited; furthermore, many acutely ill patients have poor imaging windows, making detection of LVSD by point-of-care cardiac ultrasound more challenging. An AI-ECG, on the other hand, can provide diagnostic predictions in a matter of seconds. AI-ECG may serve as a non-invasive, inexpensive, and rapid diagnostic and screening tool in such circumstances. Potential future applications could include early diagnosis of shock to allow targeted intervention, diagnosis of heart failure and prediction of decompensation, as well as differentiation of cardiogenic vs. non-cardiogenic pulmonary edema among others.

## AI-ECG For Mortality Prediction

Despite recent advances in bedside mortality prediction tools in the CICU, these models and clinical biomarkers are not without limitations, including the need to obtain blood samples and run laboratory assays. An additional limitation is the lack of integration of cardiac function markers, which may be an essential component of risk stratification in CICU patients (Jentzer et al., 2019a). The AI-ECG presumably identifies electrical signals reflecting underlying myocardial disease that may contribute to increased mortality. Therefore, AI-ECG algorithms that can predict underlying myocardial disease such as AF and LVSD may be used to construct models that aid in mortality prediction. This might then identify high-risk patients who may benefit from more invasive and extensive testing early in their CICU admission (Jentzer et al., 2021b).

Another CICU study demonstrated that an AI-ECG algorithm designed to predict LVSD could identify impacts of myopathic processes on cardiac electrical activity and predict outcomes based on these changes prior to the development of mechanical dysfunction (Jentzer et al., 2021b). This study population included 11,266 adults with a mean age of 67.6 ± 15 years and 37.3% female admitted to the Mayo Clinic Rochester CICU from January 1, 2007 to April 30, 2018. They applied the established ECG algorithm that was designed to predict the probability of LVSD to the first ECG performed after CICU admission to determine whether the probability of LVSD identified by AI-ECG was associated with clinical outcomes. They observed that a higher probability of LVSD provided by the AI-ECG was associated with an increased risk of hospital mortality and 1-year mortality even after adjustment for standard prognostic variables (Jentzer et al., 2021b). This was true even though the AI-ECG algorithm was imperfect at detecting echocardiographic LVSD. The added mortality risk associated with a higher AI-ECG LVSD probability was not entirely explained by the presence of reduced LVEF by echocardiography. Importantly, the AI-ECG had an even stronger association with mortality in patients without significant LVSD by echocardiography, implying that the AI-ECG was likely identifying subclinical myocardial disease that carried prognostic information (Jentzer et al., 2021b). A higher AI-ECG LVSD probability prediction was associated with increased risk of hospital and 1-year mortality even after stratification by echocardiographic LVEF (Jentzer et al., 2021b). Upon further analysis of the 1-year mortality prediction, patients with a true positive AI-ECG (i.e., both the AI-ECG and the TTE showed LVSD) had a higher 1-year mortality than patients with either a false negative AI-ECG or a false positive AI-ECG; patients with a true negative AI-ECG (i.e., both the AI-ECG and the TTE showed no evidence of LVSD) had the lowest mortality (Jentzer et al., 2021b). The patients with either false positive or false negative AI-ECG results, in which there was a discordance between LVSD by AI-ECG and by TTE, had similar outcomes (Jentzer et al., 2021b). This highlights the clinical relevance of the ECG features identified by the AI-ECG algorithm, which carry as much prognostic relevance as the LVEF itself and contribute incrementally to outcome prediction (Jentzer et al., 2021b). One limitation is that it is unclear which ECG features were considered significant by the AI-ECG algorithm; thus, it makes it difficult to identify potential features contributing to the AI-ECG predictions (Jentzer et al., 2021b). Considering that the AI-ECG algorithm used in this analysis was not designed to predict mortality, it is remarkable that the same ECG features that predict LVSD also predict mortality.

Other analyses have emphasized the power of an AI-ECG algorithm to predict mortality outside of the CICU setting. Raghunath et al. demonstrated that the AI-ECG was able to predict 1-year mortality even in patients with a “normal” ECG by cardiologist interpretation (Raghunath et al., 2020). This large study trained an AI-ECG algorithm on 1,169,662 resting ECGs from 253,397 patients including predominantly outpatients. The AI model used in this study was implemented using Keras with a Tensorflow backend in Python and was thoroughly trained using the sample size and then tested for validation. It was ensured that ECGs used for training were not also used for testing (Raghunath et al., 2020). The model was able to predict 1-year all-cause mortality in this population with an AUC of 0.876, even when the ECG was interpreted as “normal” by expert cardiologist interpretation (Raghunath et al., 2020). This work highlights the importance of mortality prediction and the false reassurance a normal ECG may provide clinicians regarding the presence of prognostically-important underlying cardiac disease. If an AI-ECG algorithm could perform as well for predicting mortality in CICU patients as this algorithm did in a mixed cohort, then the level of performance would meet or exceed that demonstrated for any published ICU or CICU risk score. These studies further substantiate the idea that the use of AI-ECG algorithms may improve mortality prediction.

## Limitations and Future Directions

While the advancements of artificial intelligence and its application to the CICU and specifically to mortality prediction is an ever-growing field, there are some limitations to its implementation to mortality prediction. AI-based models are heavily reliant on input data. Because there is so much input data available in the CICU, models can become compromised if the wrong data is used for model development. A thorough evaluation of potential data points must be considered prior to model development and multiple models may have to be developed to identify the optimal data points which may be time and cost consuming. An additional important aspect to consider with AI-based mortality prediction, is ability to explain the model and its prediction. Current prediction models provide an explanation for the model's predictions, allowing clinicians to trust the data that is yielding from the use of these models. While AI-based prediction models have the potential to out-perform traditional predictions, the features that contribute to the prediction of the AI-ECG model may not always be clear. This makes its methods difficult to explain and can be a hindrance to clinical adoption by practitioners.

Another potential limitation that AI-ECG has compared to traditional prediction models using risk scores is its incorporation of active clinical signs (e.g., vital signs and laboratory values) and patient symptoms in the prediction model. It is conceivable that integrating dynamic, real-time clinical and/or laboratory data with AI-ECG data could provide an even more accurate prediction of mortality, particularly if a CICU-specific mortality prediction AI-ECG model was developed. The advantage current prediction tools have over AI-ECG models is the emphasis on the patient's clinical factors such as age, severity of illness, vital signs, laboratory values, and symptoms. These are dynamic aspects of the patient's clinical presentation that are essential in predicting mortality and outcomes, yet currently available risk scores do not consistently integrate serial values to enhance risk prediction. If the AI-ECG algorithm could integrate patient clinical information in a dynamic fashion, the ability to predict mortality in the CICU may exceed what is currently available through risk score assessment. One retrospective study showed that when data from the electronic medical record and dynamic, serial monitoring of heart rate and blood pressure were applied to a high-performing prediction model using AI, sepsis could be predicted 4 h in advance (Nemati et al., 2018).

Additionally, AI-ECG algorithms could extend to incorporate continuous telemetry monitoring in CICU patients. The continuous nature of telemetry would allow the AI-ECG algorithm to interpret dynamic changes in the cardiac electrical activity throughout the patient's CICU course. Early incorporation of this concept has been shown in studies in which patterns and changes in continuous telemetry monitoring are analyzed to predict in hospital cardiac arrest. One study showed that a predictive model could be used to predict in hospital cardiac arrest and that trending telemetry changes was feasible (Do et al., 2019). Similar monitoring of telemetry changes has shown that machine learning and AI techniques can be applied to telemetry waveforms to also predict sepsis (Bravi et al., 2012). Preliminary studies have also applied AI models to predict hypotension (Chan et al., 2020). Sequential monitoring of variables used in a risk score may provide better mortality prediction than a single snapshot assessment. A similar idea can be considered with continuous telemetry monitoring with AI capabilities, whereby dynamic changes in cardiac activity can provide real-time mortality prediction updates and alerts for impending clinical events (e.g., predicting the onset of AF) that could guide management decisions.

An AI-ECG model might also be used in conjunction with the current prediction models, such as M-CARS, to better risk stratify CICU patients and predict in-hospital and post-discharge mortality. To date, no specific AI-ECG algorithm has been developed to predict mortality in CICU patients, leaving an opportunity to integrate AI-ECG data with clinical variables for risk prediction. Regarding the diagnostic performance of AI-ECG for various cardiac conditions, this needs to be meticulously compared against gold standard diagnostic criteria and re-validation may be needed in the CICU population where disease epidemiology might differ from populations used to derive models. Furthermore, diagnostic AI-ECG model performance optimization in CICU patients may require determination of population-specific cut-offs that could differ from other cohorts.

Ultimately, randomized controlled trials will be needed with the development of AI-ECG models to assess these models prospectively and compare then against currently implemented risk scores. As models begin to develop and prove useful, additional components and dynamics factors can be used to augment the prediction models.

## Conclusion

The ability to identify occult disease processes and predict patient outcomes is important in the care of CICU patients. This capability has clinical benefits that include early mortality prediction, identification of patients who may benefit from invasive testing or intervention, and identification of patient who may not need ICU-level of care. The interest in predicting outcomes in ICU settings is exemplified by the recent proliferation of prediction tools (Table 6). Although many of these tools are valuable, many lack validation in the CICU population and there is considerable variability in their performance. Various AI-ECG algorithms have demonstrated excellent predictive abilities in the CICU and may fill some of the gaps left by traditional prediction models.

**Table 6:** Summary of mentioned prediction tools.

**Prediction tool**	**Assessment population**
Braden skin score	Predict risk of skin pressure injury in admitted patients
TIMI, GRACE, and Zwolle risk scores	Developed for patients with AMI or ACS
EFFECT, OPTIMIZE, GWTG-HF	Developed for patients with acute decompensated HF
M-CARS	Mortality prediction tool designed for CICU patient populations
Jentzer et al., 2021a	Predict the risk of LVSD in CICU patients using AI-ECG
Kashou et al., 2021	Prediction of LVSD in patients with AF in the CICU using AI-ECG
Jentzer et al., 2021b	Prediction of outcomes based on prediction of LVSD using AI-ECG
Raghunath et al., 2020	Use of AI-ECG to predict mortality, not limited to the CICU patient population

## Author Contributions

NR wrote the first draft of the manuscript. JJ, PN, and AK wrote sections of the manuscript. All authors were involved in the conception and outlining of the manuscript. All authors contributed to manuscript revision, read, and approved the submitted version.

## Conflict of Interest

The authors declare that the research was conducted in the absence of any commercial or financial relationships that could be construed as a potential conflict of interest.

## Publisher's Note

All claims expressed in this article are solely those of the authors and do not necessarily represent those of their affiliated organizations, or those of the publisher, the editors and the reviewers. Any product that may be evaluated in this article, or claim that may be made by its manufacturer, is not guaranteed or endorsed by the publisher.
